# The impact of DIP payment reform on hospitalization costs and equity in type 2 diabetes mellitus patients: evidence from a pilot city in Central China

**DOI:** 10.3389/fpubh.2026.1775557

**Published:** 2026-05-04

**Authors:** Yiming Sheng, Ting Sun, Luyu Mo, Dan Wu, Xuehui Meng

**Affiliations:** School of Humanities and Management, Zhejiang Chinese Medical University, Hangzhou, China

**Keywords:** DIP payment reform, hospitalization costs, hospitalization equity, ITSA, type 2 diabetes mellitus

## Abstract

**Background:**

In order to regulate the surging medical spending, the Chinese government conducted a reform of medical insurance payment directed to regional global budgets and Diagnosis-Intervention Packet (DIP) system.

**Methods:**

An interrupted time series analysis was conducted using monthly medical insurance claims data from 45,900 T2DM inpatients in S City, China between January 2020 and December 2023, comparing outcomes before and after the January 2021 DIP reform implementation across insurance types (UEBMI vs. URRBMI) and hospital types (TCM vs. general hospitals).

**Results:**

The DIP reform significantly reduced total hospitalization costs and length of stay overall, but divergently affected out-of-pocket ratios—decreasing them for UEBMI enrollees while significantly increasing them for URRBMI patients and TCM hospital patients—indicating worsened financial equity for vulnerable subgroups despite improved cost.

**Conclusion:**

While the DIP payment reform effectively reduced hospitalization costs and length of stay for T2DM patients, it simultaneously exacerbated out-of-pocket inequities across insurance schemes and hospital types, indicating that future payment policies must integrate cost containment with health equity.

## 1 Introduction

Diabetes mellitus (DM) has remained a formidable international health enemy as it is one of the leading causes of death and disability in the globe (1, 2). The economic burden posed by type 2 diabetes mellitus (T2DM) to the healthcare systems of the world is enormous as it covers about 90–95 percent of all clinical cases (3, 4). T2DM rates grow every year due to the aging population and high levels of sedentary lifestyles (5). This financial strain has caused countries to understand the strategies of containment by cost; Taiwan, an example, has resorted to Pay-for-Performance scheme, and Singapore to the Chronic Care Model as a way of managing the disease (4, 6).

In China, CMII system has reached to almost every population compelling more than 1.3 billion lives (7, 8). However, the Fee-for-Service (FFS) model was rather ineffective and was characterized by over-treatment, waste of resources, and cost increase (9). The Diagnosis Related Groups (DRG) payment reform, implemented by the government in 2019, was made to curb this. Following the global precedent, the DRG system includes patients based on their clinical image and resource consumption with the aim to strengthen their efficiency and service delivery (10, 11).

Although this was meant to curb FFS-induced inflation, initial DRG pilots would give rise to the apprehension of healthcare equity and excellence (12, 13). This discomfort led to the development of Diagnosis-Intervention Packet (DIP) (14). Building on the DRG base, DIP uses mathematical modeling to investigate the data of diseases and treatment, producing uniform grouping in terms of reimbursement (15). Their difference is in methodology: when DRG uses expert consensus and clinical pathways to determine the way resources are used (31), DIP rotates towards the statistical mining of large real-world data (16, 17). Precisely, it bundles the diagnosis-treatment combinations, usually a diagnosis-primary procedure combination, to core payment units.

Although literature indicates that DIP has an advantage over DRG in terms of coverage of services comprehensively (18), the use of this approach has also aroused controversy on the issue of equity and heterogeneity. The effects seem to be different across hospitals, tier of the economy, and even across the type of disease (19). Importantly, much research has been done by scholars to examine the impact of DIP on high-burden, high-risk diseases (20, 21), but few studies have used systematic reviews to examine the specific outcomes of T2DM treatment.

Equity is another concept upon which Chinese researchers and policymakers have been keen to look into as they seek innovative approaches in payment models of medical insurance. China’s basic medical insurance system is characterized by marked institutional segmentation. Specifically, the financing mechanisms, benefit ceilings, and socioeconomic characteristics of enrollees all vary between the Urban Employee Basic Medical Insurance (UEBMI) and the Urban and Rural Resident Basic Medical Insurance (URRBMI). Because the DIP primarily targets medical service providers, differences in equity between beneficiaries covered by different insurance schemes were not explicitly incorporated into its initial design. Accordingly, we divided the full sample into two subgroups according to insurance type-UEBMI and URRBMI-to examine heterogeneity by scheme.

The second source of heterogeneity is hospital type. Many studies have explored how the level of hospitals influences the effectiveness of DIP reform (22–24); while a comparison between Traditional Chinese Medicine (TCM) hospitals and general hospitals remains scarce. The underlying logic of disease classification and coding in DIP is deeply rooted in the modern medicine system of disease classification and standardized treatment procedures. TCM, in its turn, rests on its version of traditional medicine theory and has distinct treatment methods, such as acupuncture and Tuina. TCM provides superior emphasis on syndrome differentiation and holistic thinking, which results in the very individualized treatment that cannot be easily translated into a single diagnosis and procedure code. However, there is some evidence that TCM could be beneficial in the treatment of T2DM (25, 26, 32). Therefore, it is interesting to investigate whether this new form of payment has different implications on hospitals in different types.

This paper chose S City, a prefecture level city in China central and one of the earliest pilot cities as the study area. We focused on patients with T2DM in S City to estimate the effect of the DIP across hospital types and basic medical insurance schemes. By investigating the reform impacts across different medical systems, this study will contribute to filling the gap in the current literature on how DIP reform affects the outcomes of patients with T2DM. The findings may help further research in improving medical insurance fund use efficiency, alleviating the out-of-pocket burden of patients, and promoting fairness and efficiency in the allocation of medical resources.

## 2 Methods

### 2.1 Study design

S City is a prefecture-level city in Hubei Province, Central China. S City was listed as an initial DIP pilot city in 2021 and officially carried out the reform at the beginning of 2021. It has been 2 years since then, and the DIP reform of the city has achieved apparent progress and won recognition from both the National Healthcare Security Administration and the Provincial Healthcare Security Administration; thus, it has participated in the national demonstration for DIP piloting. Given the leading role of S City in this reform, we adopt administrative data from the Healthcare Security Administration of S City for our empirical analysis.

S City operates three municipal districts, one county-level city, four counties, one economic development zone, and one tourism special zone. In 2024, the permanent resident population of the city was 3.1422 million. The total GDP of S City reached 256.58 billion RMB, ranking 139th in the country, which signifies the city has a medium level of economic development and is a typical prefecture-level city in China. In the same year, the number of enrollees in the UEBMI and the URRBMI was 0.55 million and 2.44 million, respectively, with an overall medical insurance coverage rate above 95%.

By the end of 2021, S City had expanded DIP to all 10 counties (cities and districts), with coverage at all hospital levels and all inpatient disease categories. In order to exclude the impact of other policies that might have affected inpatient service utilization, we comprehensively reviewed the published literature and relevant policy documents to identify and eliminate confounding variables. Given the advantages of Interrupted Time Series (ITS) in policy evaluation, ITS analysis was adopted as the main research method. First, the DIP reform covered the whole city, so a natural control group was unavailable. Second, the ITS method estimates intervention effects by comparing trends before and after policy implementation, thus it can effectively distinguish the policy impact from seasonal variation. Third, ITS models have been widely applied in studies assessing DRG and DIP reforms and are well supported in health policy research (27, 28). In light of relevant policy timelines, January 2021 was defined as the time point for intervention, and the ITS model was adopted to investigate the effect of the DIP reform on the outcomes of hospital services among different insurance types and different types of hospitals in S City.

### 2.2 Data sources and samples

As no major outbreaks of COVID-19 were reported in S City, the disruption in medical service delivery was low during the pandemic period, thus facilitating reliable time series analysis. In this paper, monthly medical insurance claims data are used for the empirical analysis from January 2020 to December 2023, extracted from the Medical Insurance Information Platform of S City. All data had been anonymized in order to protect the privacy of patients.

Each claim record contained information on patient age, sex, admission and discharge dates, insurance type, total hospitalization costs, OOP costs, and the medical institution. During data cleaning, records unrelated to general inpatient hospitalizations were excluded. To avoid double counting due to cross-year hospitalizations, cases spanning calendar years within the observation period (admission in 2020 and discharge in 2021) were counted only once according to the admission date. In addition, because this study focused on hospitalization events occurring within the study period, only records with both admission and discharge dates falling between January 2020 and December 2023 were included. The final analytical sample comprised 45,900 patients.

#### 2.2.1 Type 2 diabetes

T2DM is a prevalent chronic metabolic disorder characterized by insulin resistance and relative insulin deficiency, primarily manifested as persistent hyperglycemia. Poor glycemic control results in serious acute and chronic complications, such as HHS and diabetic nephropathy. Currently, one of the major causes of death in the world is diabetes.

Management strategies for T2DM include lifestyle modification and pharmacotherapy. For adult patients with obesity-related T2DM who respond poorly to medical treatment, metabolic surgery may be considered. Given the large size of the T2DM population and the limited influence of environmental factors, such as seasonal variation, on disease occurrence, this study selected hospitalization records identified using the International Classification of Diseases, Tenth Revision (ICD-10) code E11. These records were retained in the final analytical dataset after data cleaning.

#### 2.2.2 Types of health insurance

Based on demographic coverage and structural frameworks, China’s medical security system is primarily bifurcated into the Urban Employee Basic Medical Insurance (UEBMI) and the Urban and Rural Resident Basic Medical Insurance (URRBMI). The UEBMI functions as a compulsory program specifically for the urban workforce. Financed through dual contributions from both enterprises and staff, this scheme focuses on reimbursing costs related to inpatient hospitalization and outpatient treatments. In sharp contrast, the URRBMI targets rural residents, urban non-employed residents, and students. As a government-initiated yet voluntarily enrolled scheme, it is funded through a combination of individual contributions and government subsidies and primarily covers inpatient services, along with a limited share of outpatient expenses.

#### 2.2.3 Hospital type

In terms of academic specialization, hospitals in China are broadly classified into four categories: general hospitals, TCM hospitals, institutions practicing integrated TCM and Western medicine, and ethnic hospitals. It should be noted that ethnic hospitals were omitted from the scope of the current research. In this current study, the category of general hospitals includes exclusively Western medicine hospitals, while the category of TCM hospitals encompasses both TCM hospitals and hospitals practicing combined traditional Chinese and Western medicine. Such categorization is justified by large differences in service models and diagnostic-therapeutic philosophies across these hospital types.

General hospitals are mainly based on the pathophysiological framework of modern medicine, while pharmacotherapy and surgery are major treatment modalities. However, the medical practice in TCM hospitals including TCM and integrated hospitals is mainly guided by the theories of TCM and characteristically emphasizes TCM interventions, such as herbal medicine, acupuncture, and Tuina, as main therapeutic approaches. While integrated traditional Chinese and Western medicine hospitals use the techniques of modern medicine, the characteristic integrative application of the principles of TCM defines them and thus essentially differentiates these from general hospitals.

### 2.3 Measurement variables

The primary objective of this research was to evaluate how the DIP reform influenced healthcare costs, service quality, and the variations existing across different insurance plans and hospital categories. Consequently, total hospitalization expenses (THC), Length of Stay (LOS), and the Out-of-pocket (OOP) ratio were selected as the key dependent variables for this analysis. For controlling the impacts of inflation and other macroeconomic factors, total hospitalization costs were adjusted using the annual Consumer Price Index (CPI) of S City, taking 2020 as a base year, so that the estimates reflected real changes in medical expenditures. Considering the fact that cost data usually have a right-skewed distribution, the natural logarithm of total hospitalization cost was adopted to enhance normality and reduce extreme values. The LOS in days refers to the difference between admission and discharge dates, and the OOP ratio is defined as the proportion of OOP expenditure relative to total hospitalization cost.

### 2.4 Statistical analysis

Data processing was conducted using SPSS software (version 24.0). Continuous variables were expressed as means $\pm$ standard deviations, while categorical variables were presented as percentages. To assess differences in baseline characteristics and outcomes between the pre- and post-reform phases, we utilized Student’s *t*-tests for continuous data and chi-square tests for categorical data. Furthermore, an Interrupted Time Series (ITS) model was implemented to quantitatively evaluate the DIP reform’s influence on total hospitalization expenses, LOS, and the OOP ratio.

January 2021 was designated as the intervention start, and monthly measurements across 48 time points were used as the analytic unit. The ITS model was formulated as:


$$Y_{t}=\beta_{0}+\beta_{1}T_{t}+\beta_{2}\mathrm{DI}P_{t}+\beta_{3}\mathrm{DI}P_{t}T_{t}+\varepsilon_{t}$$

In this equation, $Y_{t}\;$ represents the outcome at month t (total hospitalization cost, LOS, or OOP ratio). $\beta_{0}\;$ denotes the initial level at the beginning of the study window, and $\beta_{1}$ reflects the underlying pre-intervention temporal trend. $\beta_{2}$ estimates the immediate level shift associated with introduction of the DIP reform, while $\beta_{3}$ captures the change in slope after the intervention compared with the pre-reform trend; thus, the post-intervention trend is given by $\beta_{1}$ . + $\beta_{3}$ . $T_{t}$ i is a continuous time index spanning the entire observation period. $\mathrm{DI}P_{t}$ is a dichotomous indicator of the reform (coded 0 prior to January 2021 and 1 thereafter). The interaction term $\mathrm{DI}P_{t}T_{t}$ epresents time since the reform was implemented. $\varepsilon_{t}\;$ is the stochastic error term.

To address potential autocorrelation, regressions were first estimated using the official *itsa* command without autocorrelation correction. Autocorrelation was then assessed using the *actest* command, with particular attention to first- and second-order autocorrelation. When first-order autocorrelation was detected, the *prais* option was applied for correction. In cases of second-order autocorrelation, the *lag (2)* option was incorporated into the model.

## 3 Results

### 3.1 Sample characteristics

**Figure 1:**
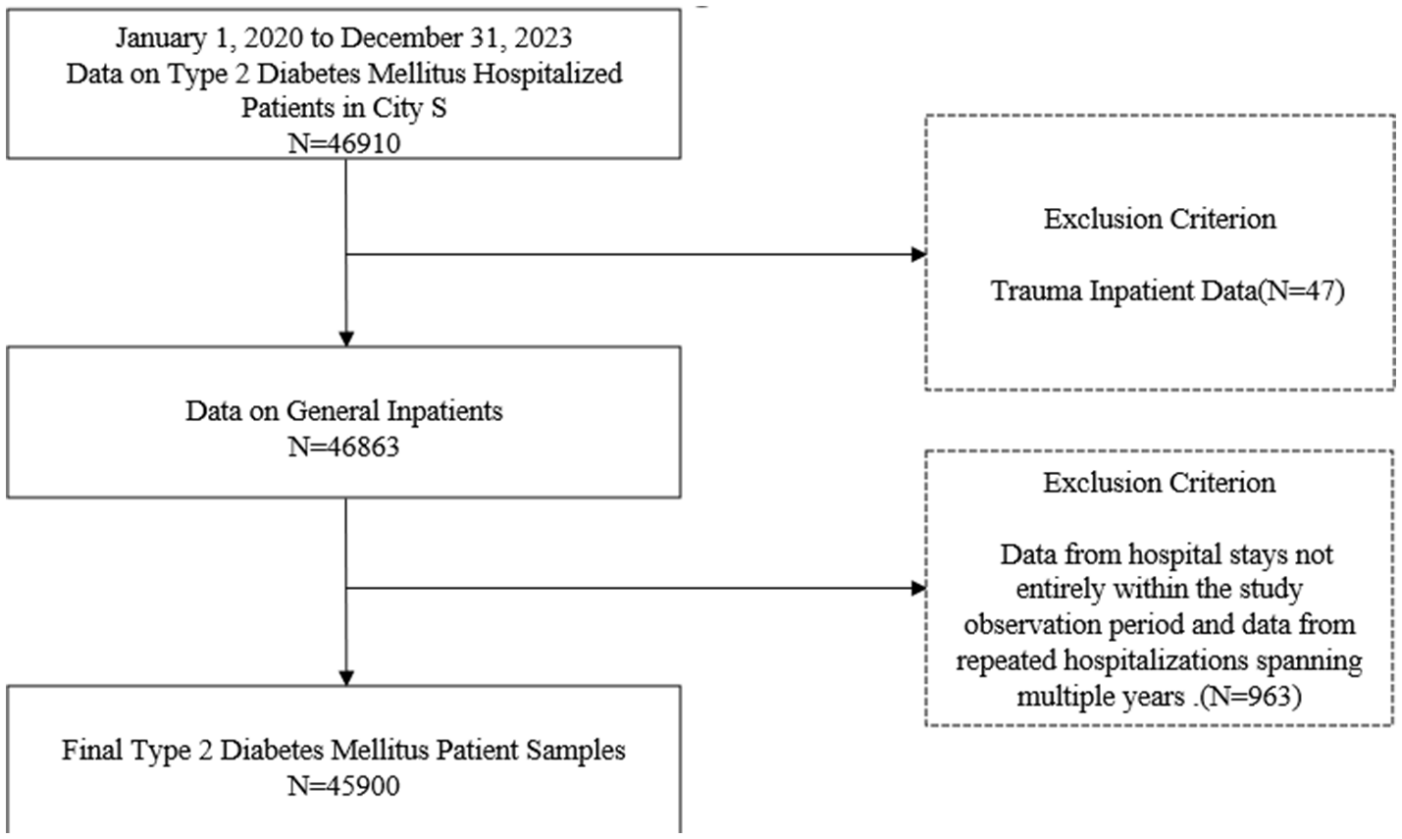
Schematic diagram of attrition in the study population.

Table 1 presents a comparative analysis of baseline demographics and outcome metrics for patients with T2DM across the pre- and post-DIP reform phases. We performed t-tests on age, THC, LOS, and OOP ratio before and after the reform, and chi-square tests on gender, insurance type, and hospital type. All results were statistically significant at the 0.1% level, with the exact significance levels in Table 1. Notably, the mean age of the cohort rose significantly (*p* = 0.000) from 58.88 years before the DIP payment reform (2020) to 59.52 years after the reform (2021–2023). However, the absolute difference was only 0.96 years, which is of limited clinical significance. Therefore, no adjustment for age was made in this study. Before the reform, there were 2,027 and 4,800 patients covered by UEBMI and URRBMI, respectively; this increased to 13,911 and 25,162 after the reform. As for hospital type, there were 948 treated in TCM hospitals and 5,879 treated in general hospitals before the reform, compared to 4,801 and 34,272 in the post-reform period, respectively. For medical expenditure, the mean total hospitalization cost per patient significantly decreased (*p* = 0.000, 95% CI: 768.15, 1061.54) from 6,949.81 RMB before the reform to 6,035.65 RMB after the reform. Meanwhile, although the change in the average length of stay (LOS) was statistically significant (*p* = 0.00, 95%CI: 0.66, 0.90), the mean LOS before and after the reform was 9.93 days in both periods, which is of no clinical significance. In contrast, the OOP ratio significantly increased (*p* = 0.000, 95%CI: −0.013, −0.006) from 29.59% before the reform to 30.54% after the reform (Figure 1).

**Table 1:** Characteristics of type 2 diabetes mellitus patients in City S from 2020 to 2023.

Variables	Before DIP reform		After DIP reform				*p-*value
	Year	Total	Year			Total	
	2020		2021	2022	2023		
Discharge cases, No.	6,827	6,827	9,852	12,866	16,355	39,073	
Age, mean (SD)	58.88 (11.69)	58.88 (11.69)	59.00 (11.76)	59.52 (11.75)	59.84 (11.60)	59.52 (11.70)	0.000
Sex, No. (%)							0.000
Male	3,173 (46.48)	3,173 (46.48)	4,762 (48.34)	6,394 (49.70)	8,194 (50.10)	19,350 (49.52)	
Female	3,654 (53.52)	3,654 (53.52)	5,090 (51.66)	6,472 (50.30)	8,161 (49.90)	19,723 (50.48)	
Medical insurance, No. (%)							0.000
Urban employee basic medical insurance	2027 (29.69)	2027 (29.69)	3,411 (34.62)	4,534 (35.24)	5,966 (36.48)	13,911 (35.60)	
Urban and rural resident basic medical insurance	4,800 (70.31)	4,800 (70.31)	6,441 (65.38)	8,332 (64.76)	10,389 (63.52)	25,162 (64.40)	
Hospital type, No. (%)							0.000
Traditional Chinese medicine hospital (*N* = 10)	948 (13.89)	948 (13.89)	1,295 (13.14)	1,596 (12.40)	1910 (11.68)	4,801 (12.29)	
General hospital (*N* = 171)	5,879 (86.11)	5,879 (86.11)	8,557 (86.86)	11,270 (87.60)	14,445 (88.32)	34,272 (87.71)	
Total hospitalization cost, mean (SD), RMB	6949.81 (5852.20)	6949.81 (5852.20)	6805.48 (5525.99)	5918.72 (4714.22)	5663.91 (4239.32)	6035.65 (4770.93)	0.000
Length of stay, mean (SD), day	9.93 (4.86)	9.93 (4.86)	9.67 (4.66)	9.23 (4.33)	8.76 (3.70)	9.93 (4.18)	0.000
Out-of-pocket ratio, mean (SD), %	29.59 (14.11)	29.59 (14.11)	29.49 (13.62)	30.63 (13.33)	31.10 (13.45)	30.54 (13.47)	0.000

### 3.2 Total hospitalization cost

**Figure 2:**
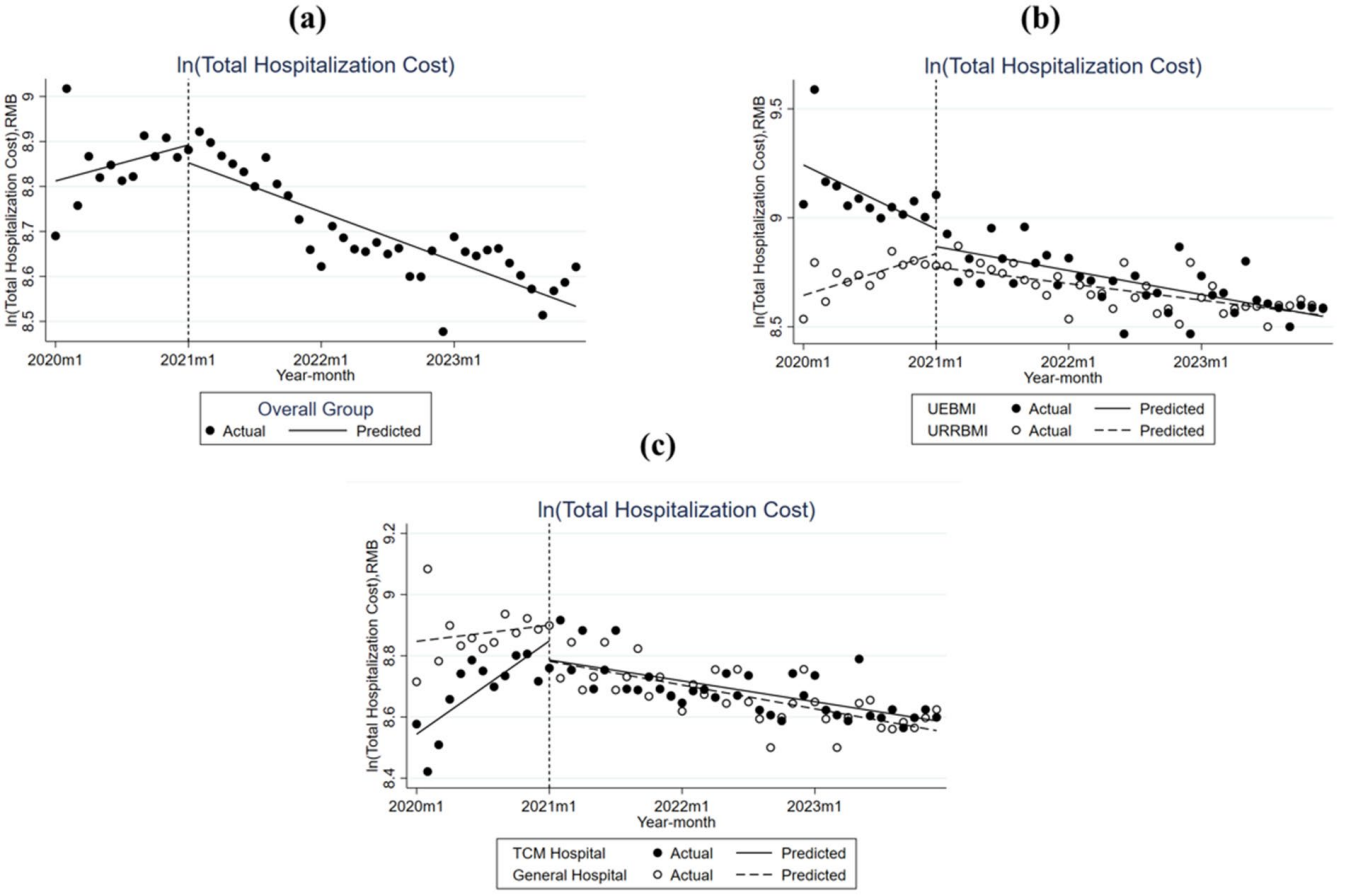
Monthly trend of total hospitalization cost (THC) per Type 2 Diabetes Mellitus inpatient case in City S. **(a)** Overall group; **(b)** Urban Employee Basic Medical Insurance (UEBMI) and Urban and Rural Resident Basic Medical Insurance (URRBMI); **(c)** Traditonal Chinese Medicine (TCM hospital) and general hospital.

As illustrated in Figure 2b and Table 2, there were significant differences in the baseline levels between patients covered by UEBMI and URRBMI before the reform, while both groups showed an overall decrease in total hospitalization costs after the reform. For patients covered by UEBMI, the hospitalization cost at baseline was 9.242, which decreased by 0.025 every month before the reform. After the implementation of the policy, the post-intervention trend increased significantly by 0.015 per month from the pre-intervention trend. In contrast, for the patients covered by URRBMI, the baseline cost was 8.644, with a significant monthly increase of 0.016 before the reform. After the DIP reform, the trend declined significantly by 0.022 per month compared to that before the reform.

**Table 2:** ITSA analysis results of total hospitalization cost (THC).

Ln (total hospitalization cost)	Category	Baseline monthly slope (*β*1)	Step change (*β*2)	Monthly slope change (*β*3)	Constant (*β*0)
		Estimate (95%CI)	Estimate (95%CI)	Estimate (95%CI)	Estimate (95%CI)
	Overall group	0.007 (−0.011, 0.024)	−0.040 (−0.123, 0.044)	−0.016 (−0.033, 0.002)*	8.812 (8.676, 8.948)***
	UEBMI	−0.025 (−0.035, −0.014)***	−0.080 (−0.142, −0.019)	0.015 (0.005, 0.026)***	9.242 (9.116, 9.369)***
	URRBMI	0.016 (0.008, 0.024)***	−0.062 (−0.122, −0.001)**	−0.022 (−0.030, −0.014)***	8.644 (8.540, 8.748)***
	TCM hospital	0.025 (0.013, 0.037)*	−0.062 (−0.118, − 0.007)	−0.031 (−0.043, −0.019)*	8.544 (8.451, 8.636)***
	General hospital	0.004 (−0.015, 0.024)	−0.118 (−0.213, −0.023)**	−0.011 (−0.030, 0.008)	8.847 (8.695, 9.000)***

*p < 0.1; **p < 0.05; ***p < 0.01.

It can also be further inferred from Figure 2c and Table 2 that the medical expenses for T2DM patients treated in different types of hospitals, namely TCM hospitals and general hospitals, generally decreased after the reform. For TCM hospitals, the hospitalization cost at baseline was 8.544, with a monthly increment of 0.025 before reform; after implementation, the trend had a further decline of 0.031 every month compared with the trend before reform. In general hospitals, the baseline cost was 8.847, and the hospitalization costs slightly increased by 0.004 every month before the reform. The post-reform trend saw a decline of 0.011 every month compared with the pre-intervention trend, but this did not attain statistical significance.

### 3.3 Length of stay

**Figure 3:**
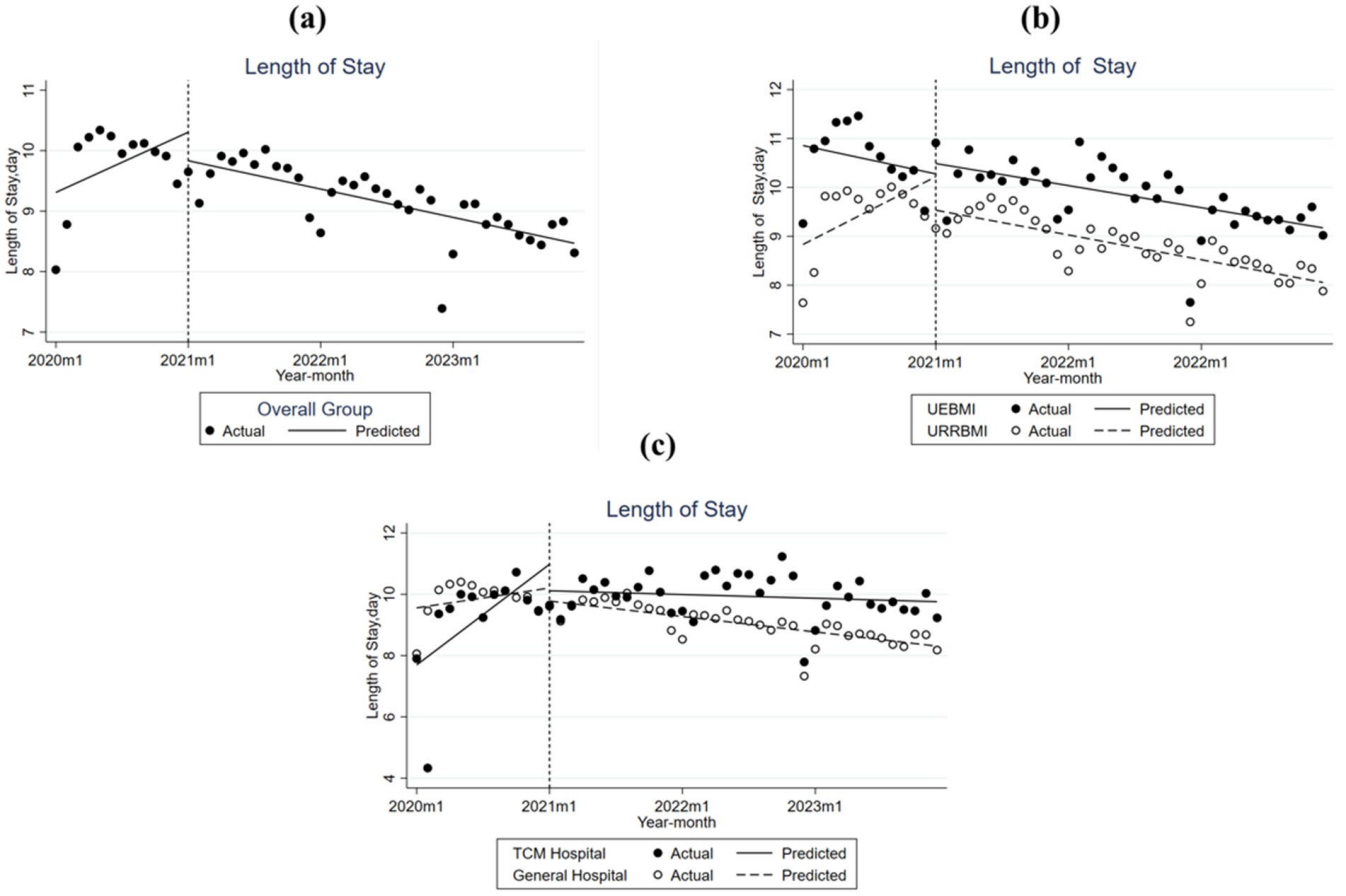
Monthly trend of length of stay (LOS) per Type 2 Diabetes Mellitus inpatient case in City S. **(a)** Overall group; **(b)** UEBMI and URRBMI; **(c)** TCM hospital and general hospital.

Trends in average LOS among patients with T2DM in S City, across different subgroups before and after the implementation of the DIP reform, are shown in Figure 3 and Table 3. Overall, there was a decline in LOS post-reform, although the change was not statistically significant.

**Table 3:** ITSA analysis results of length of day (LOS).

Length of stay	Category	Baseline monthly slope (*β*1)	Step change (*β*2)	Monthly slope change (*β*3)	Constant (*β*0)
		Estimate (95%CI)	Estimate (95%CI)	Estimate (95%CI)	Estimate (95%CI)
	Overall group	0.083 (−0.068, 0.234)	−0.116 (−0.276, 0.043)	−0.472 (−1.328, 0.384)	9.309 (8.180, 10.438)***
	UEBMI	−0.048 (−0.147, 0.050)*	0.212 (−0.362, 0.785)	0.011 (−0.088, 0.110)*	10.856 (10.134, 11.577)***
	URRBMI	0.115 (−0.015, 0.245)*	−0.684 (−1.424, 0.057)*	−0.158 (−0.288, −0.027)**	8.833 (7.874, 9.791)***
	TCM hospital	0.274 (0.120, 0.428)	−0.864 (−1.671, −0.057)	−0.284 (−0.438, −0.130)	7.693 (6.516, 8.870)
	General hospital	0.054 (−0.084, 0.192)	−0.426 (−1.196, 0.345)	−0.096 (−0.235, 0.042)	9.556 (8.544, 10.568)***

*p < 0.1; **p < 0.05; ***p < 0.01.

As can be seen from Figure 3b and Table 3, there were significant differences in baseline LOS between UEBMI and URRBMI patients before the reform, while the overall trend of LOS for both groups declined after implementation. For UEBMI patients, the baseline LOS was 10.856 days, with a monthly decrease of 0.048 days before the reform. At the time of the reform, there was an immediate, insignificant increase of 0.212 days, followed by a post-reform upward trend of 0.011 days per month. In contrast, for URRBMI patients, the baseline was 8.883 days, with a pre-reform monthly increase of 0.115 days. Following the introduction of the DIP reform, we observed an instantaneous reduction of 0.684 days, coupled with a sustained monthly decrease of 0.158 days.

As shown in Figure 3c and Table 3, the trends in LOS varied by hospital type, with significant differences in baseline levels. Specifically, before the reform, the baseline LOS in TCM hospitals was 7.693 days, which was shorter compared to the 9.556 days observed in general hospitals. From visual inspection, the trends appear to indicate a decline in LOS for patients treated in both TCM and general hospitals after the reform; however, the estimated coefficients were not statistically significant.

### 3.4 Out-of-pocket ratio

**Figure 4:**
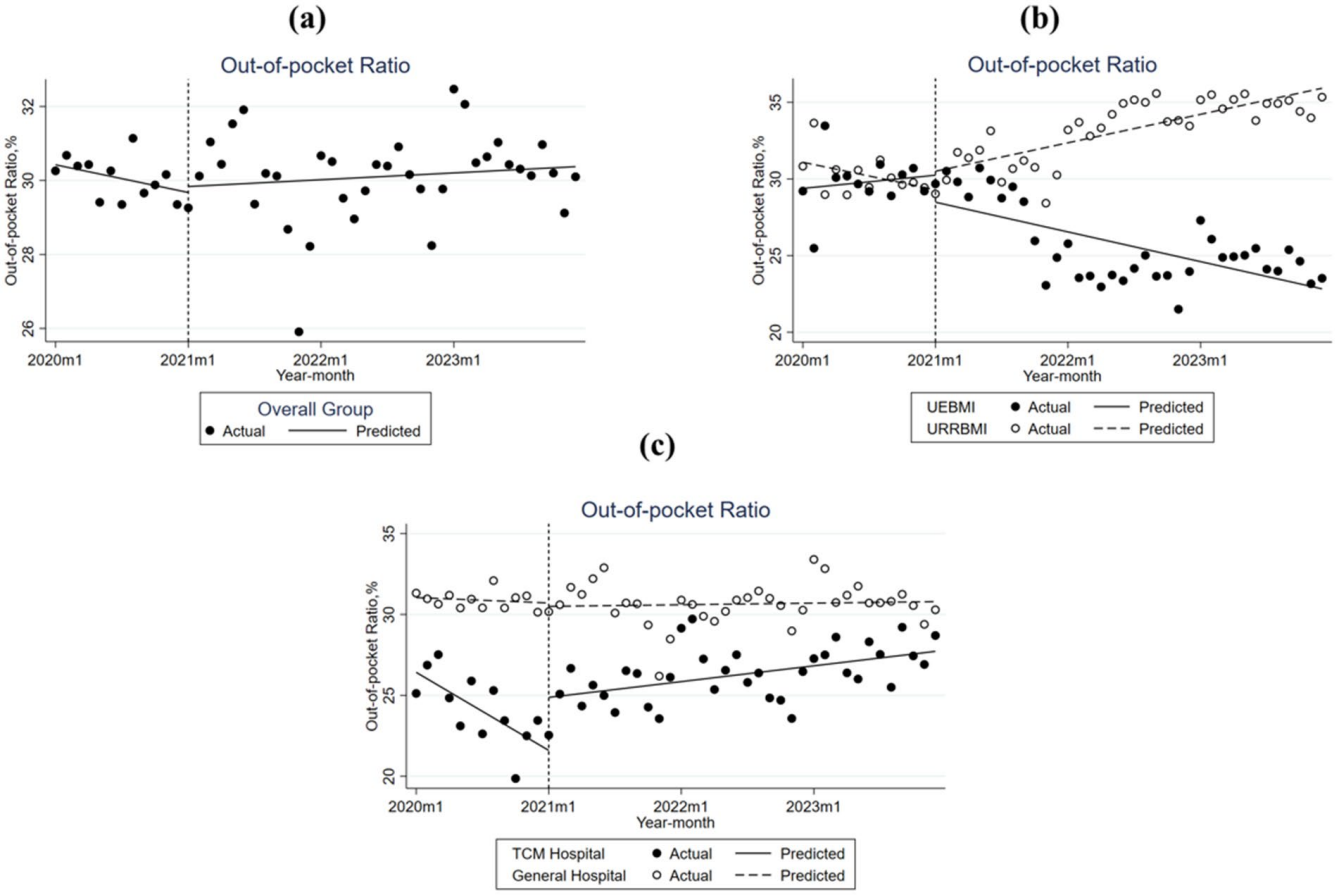
Monthly trend of out-of-pocket ratio (OOP ratio) per Type 2 Diabetes Mellitus inpatient case in City S. **(a)** Overall group; **(b)** UEBMI and URRBMI; **(c)** TCM hospital and general hospital.

Figure 4 and Table 4 illustrate the changes in the OOP ratio among patients with T2DM in S City across different subgroups before and after implementation of the DIP reform. In general, the OOP ratio shifted from a decreasing trend before the reform to an increasing trend after the implementation of the reform.

**Table 4:** ITSA analysis results of out-of-pocket ratio (OOP ratio).

Out-of-pocket ratio	Category	Baseline monthly slope (*β*1)	Step change (*β*2)	Monthly slope change (*β*3)	Constant (*β*0)
		Estimate (95%CI)	Estimate (95%CI)	Estimate (95%CI)	Estimate (95%CI)
	Overall group	−0.062 (−0.120, −0.005)**	0.161 (−0.851, 1.172)	0.078 (0.011, 0.145)**	30.424 (30.066, 30.781)***
	UEBMI	0.072 (−0.058, 0.202)*	−1.762 (−3.068, −0.455)**	−0.233 (−0.370–0.097)***	29.384 (28.289, 30.479)***
	URRBMI	−0.149 (−0.286, −0.013)**	−0.009 (−0.046, 2.455)*	0.304 (0.160, 0.448)***	31.089 (29.888, 32.289)***
	TCM hospital	−0.402 (−0.496, −0.308)***	3.278 (2.147, 4.408)***	0.483 (0.390, −0.577)***	26.422 (25.877, 26.966)***
	General hospital	−0.028 (−0.073, 0.017)	−0.211 (−1.461, 1.040)	0.036 (−0.030, 0.103)***	31.045 (30.783, 31.307)***

*p < 0.1; **p < 0.05; ***p < 0.01.

**Figure 5:**
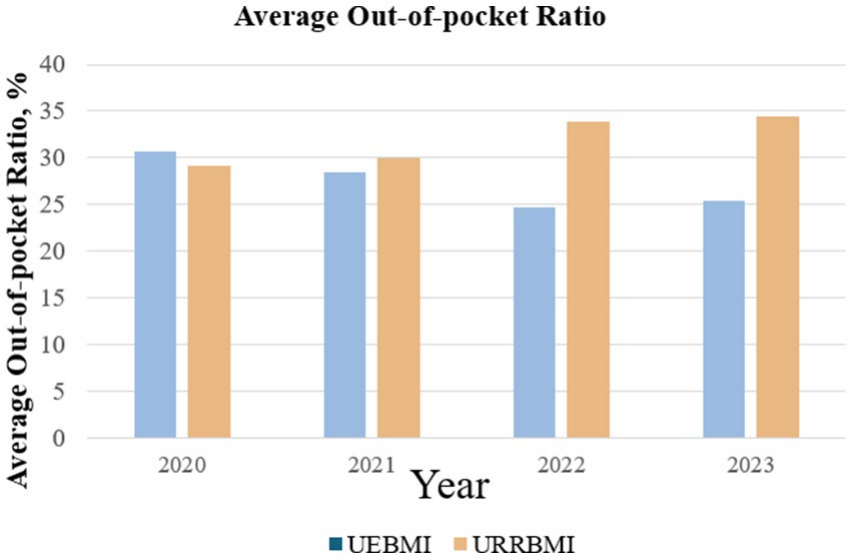
Comparison of changes in annual average out-of-pocket ratios between the UEBMI and URRBMI groups.

As presented in Figure 4b and Table 4, there were significant differences in baseline OOP ratios between patients covered by UEBMI and URRBMI before the reform. After the reform, the trend in the OOP ratio for UEBMI changed from increasing to decreasing, while that for URRBMI changed from decreasing to increasing. This may affect the equity of health insurance. For example, Zhang San, a UEBMI participant, was hospitalized for T2DM. Before the reform, his total hospitalization cost was 10,000 RMB, with OOP expenses of 2,000 RMB and reimbursement of 8,000 RMB. After the reform, his total cost was 9,000 RMB, with OOP expenses of 1,500 RMB and reimbursement of 7,500 RMB. In contrast, Li Si, a URRBMI participant, was also hospitalized for T2DM. Before the reform, his total cost was also 10,000 RMB, with OOP expenses of 2,500 RMB and reimbursement of 7,000 RMB. After the reform, his total cost was 9,200 RMB, with OOP expenses of 2,700 RMB. That is to say, if the reduction in total costs for URRBMI participants is smaller than the increase in their out-of-pocket expenses, the reform may actually harm the interests of URRBMI participants. Figure 5 also illustrates the substantial differences in the out-of-pocket (OOP) ratio between the UEBMI and URRBMI groups from 2020 to 2023. For patients covered by UEBMI, the baseline OOP ratio was 29.384%, increasing by 0.072% every month before the reform. The reform was associated with an immediate drop of 1.762%, followed by a significant drop in the post reform trend of 0.233% per month relative to the preintervention trend. In contrast, the baseline OOP ratio for patients covered by URRBMI was 31.089%, which was reduced monthly by 0.149% before the reform. The immediate policy effect was minimal, with a decrease of 0.009%, whereas the post-reform trend increased significantly by 0.304% per month compared with the pre-reform trend.

Figure 4c and Table 4 both show that OOP ratios for patients treated in different hospital types increased after the DIP reform, although the baseline levels were significantly different. The trend for OOP ratios in TCM hospitals changed from a decline to an increase after the reform. Specifically, the baseline OOP ratio was 26.422%, decreasing significantly at a rate of 0.402% every month before the reform. The policy intervention resulted in a significant immediate increase of 3.278%, and the post-reform trend increased significantly by 0.483% every month relative to the pre-reform trend. In general hospitals, the baseline OOP ratio was 31.045%, and the trend had a slight monthly decrease of 0.028% before the reform. Although the immediate effect was a small decrease of 0.211%, the post-reform trend increased, though small, but significantly by 0.036% every month compared to the pre-intervention trend.

### 3.5 Robustness tests

To rule out the possibility that outliers skewed the baseline regression results, we conducted a series of robustness checks, with full results reported in Tables 5–7. Specifically, we applied bilateral winsorization at the 5% level to the three dependent variables (total hospitalization cost, LOS, and OOP ratio) to mitigate the influence of extreme values. The models were then re-estimated using the winsorized dataset.

**Table 5:** Robustness tests of ITSA for hospitalization cost variables.

Ln (total hospitalization cost)	Category	Baseline monthly slope (*β*1)	Step change (*β*2)	Monthly slope change (*β*3)	Constant (*β*0)
		Estimate (95%CI)	Estimate (95%CI)	Estimate (95%CI)	Estimate (95%CI)
	Overall group	0.019 (0.012, 0.026)***	−0.068 (−0.119, −0.017)***	−0.025 (−0.032, −0.018)***	8.577 (8.519, 8.635)***
	UEBMI	0.003 (−0.001, 0.008)***	−0.066 (− 0.086, −0.046)	−0.010 (−0.045, −0.024)***	8.880 (8.517, 9.243)***
	URRBMI	0.020 (0.012, 0.029)***	−0.039 (−0.087, 0.009)	−0.027 (−0.036, −0.018)***	8.472 (8.403, 8.541)***
	TCM hospital	0.005 (−0.004, 0.013)*	−0.001 (−0.047, 0.045)**	−0.006 (−0.014, 0.003)**	8.602 (8.534, 8.670)
	General hospital	0.021 (0.010, 0.031)***	−0.077 (−0.113, −0.024)**	−0.028 (−0.038, −0.017)***	8.576 (8.493, 8.660)***

*p < 0.1; **p < 0.05; ***p < 0.01.

**Table 6:** Robustness tests of ITSA for hospitalization length variables.

Length of stay	Category	Baseline monthly slope (*β*1)	Step change (*β*2)	Monthly slope change (*β*3)	Constant (*β*0)
		Estimate (95%CI)	Estimate (95%CI)	Estimate (95%CI)	Estimate (95%CI)
	Overall group	0.086 (−0.029, 0.201)	−0.481 (−1.112, 0.157)	−0.115 (−0.233, 0.002)*	9.264 (8.400, 10.128)***
	UEBMI	0.214 (−0.005, 0.131)	−0.205 (−0.734, 0.324)	−0.064 (−0.156, 0.028)	8.880 (9.324, 10.694)
	URRBMI	0.090 (−0.012, 0.193)*	−0.516 (−1.050, 0.019)*	−0.125 (−0.229, − 0.020)**	9.026 (8.234, 9.818)***
	TCM hospital	0.274 (0.120, 0.307)	−0.863 (−1.380, −0.345)	−0.218 (−0.313, −0.123)	8.361 (7.646, 9.076)
	General hospital	0.065 (−0.048, 0.179)	−0.392 (−1.027, 0.242)	−0.098 (−0.214, 0.019)*	9.381 (8.516, 10.246)***

*p < 0.1; **p < 0.05; ***p < 0.01.

**Table 7:** Robustness tests of ITSA for out-of-pocket ratio variables.

Out-of-pocket ratio	Category	Baseline monthly slope (*β*1)	Step change (*β*2)	Monthly slope change (*β*3)	Constant (*β*0)
		Estimate (95%CI)	Estimate (95%CI)	Estimate (95%CI)	Estimate (95%CI)
	Overall group	−0.063 (−0.203, 0.077)	−0.087 (−1.167, 0.993)	0.101 (−0.042, 0.224)	30.180 (29.131, 31.229)***
	UEBMI	0.103 (−0.027, 0.234)**	−1.516 (−2.603, −0.429)**	−0.226 (−0.360, −0.092)***	28.837 (27.7711, 29.903)**
	URRBMI	−0.171 (−0.360, 0.019)*	0.682 (−0.701, 2.065)	0.335 (0.141, 0.528)***	31.042 (29.483, 32.601)***
	TCM hospital	−0.265 (−0.393, −0.136)*	2.684 (1.451, 3.917)**	0.375 (0.390, −0.577)***	25.801 (25.016, 26.586)***
	General hospital	−0.038 (−0.172, 0.097)	−0.438 (−1.559, 0.724)	0.064 (−0.075, 0.202)	30.809 (29.884, 31.734)***

*p < 0.1; **p < 0.05; ***p < 0.01.

As shown in the Appendices, the coefficient estimates were largely consistent with the baseline results in terms of magnitude, direction, and overall statistical significance, supporting the robustness of the main findings. Although the statistical significance of several coefficients changed slightly after winsorization, these changes were small and are expected when extreme observations are down-weighted. For example, the immediate policy effect coefficient *β*_2_ for total hospitalization cost in the URRBMI group became more statistically significant, whereas the post-reform trend coefficient *β*_3_ for LOS in the UEBMI group showed a marginal reduction in significance. On the whole, these findings signify the fact that the initial conclusions are consistent and sound.

## 4 Discussion

Based on the hospitalization data of the patients with T2DM in S City between the year 2020 and 2023, we analyzed the seen effects of the DIP reform. Overall, the total hospitalization cost and LOS decreased following the implementation. These reductions were seen in schemes of insurance and type hospital indicating better spending management and efficiency of inpatients (33, 34). Nevertheless, adjustments in the OOP ratio were more sustained and did not reflect the positive tendencies in cost and LOS.

Before the reform, the heterogeneity of hospitals in terms of levels, clinical practice, and cost designs could have been relevant to the discrepancy in the pricing and management strategies among the providers. Conversely, DIP makes use of region-specific historical information and implements a more standardized incentive design, which can coordinate providers economic incentives and induce them to behave in the same way and contain their cost control behavior across environments, whether the hospitals have majority of beneficiaries of UEBMI program with generous benefits or have a majority of the beneficiaries of URRBMI program with less generous benefits.

The decline in LOS indicates increased efficiency of the inpatients. Interestingly, the days of stay of the URRBMI patients were shorter compared to the UEBMI patients, in line with the past research (29, 30). The difference in benefit generosity and reimbursement ceilings of the two insurance schemes is one of the probable reasons. By granting longer stay times, under DIP, the margins can be lessened; therefore, the hospitals working with URRBMI patients can implement more strict discharge policy after the minimum clinical safety requirements are fulfilled. On the other hand, the increased financial latitude with the UEBMI might allow more extensive diagnostic and treatment services that have the potential to protract the LOS (36, 37).

Variations based on hospital type are also a sign of possible structural limitations. The decrease in LOS was further experienced in general hospitals but there was a slight decline in TCM hospitals. This trend might indicate an inappropriateness of DIP incentive models working with the specifics of the traditional Chinese medicine (TCM) care (e.g., acupuncture and herbal decoctions), which is per time-intensive, labor-intensive, and individualized and would tend to limit the possibility to reduce LOS without influencing continuity or quality of care.

Notably, even though the total cost and LOS fell, overall OOP ratio rose by a little upon the reform, and changes varied across insurance schemes: among UEBMI beneficiaries it went down, whereas among URRBMI patients, it grew significantly. This divergence may reflect provider’s responses to stricter conditions, differences in response to the reimbursement limit of URRBMI, and possible cost shifting to non-reimbursable services, as well as policymaking, including the increased deductibles in certain regions of fiscal pressure (38). Moreover, since UEBMI usually has a larger reimbursement catalog than URRBMI, the beneficiaries of UEBMI might get more benefits in the situations when the total expenses drop, and patients under URRBMI can get fewer advantages.

The growth in OOP ratio seemed deeper in TCM hospitals. Potential explanations include greater reliance on services outside the reimbursement catalog, frequent use of proprietary Chinese medicines that are often classified as Class B drugs with lower reimbursement rates, and the limited applicability of Volume-Based Procurement to herbal products due to standardization challenges, which may constrain cost reductions in the TCM sector (39).

This study benefits from comprehensive hospitalization records obtained from the S City Healthcare Security Administration. By conducting separate ITS analyses stratified by insurance scheme and hospital type, we were able to estimate subgroup-specific policy effects more precisely and provide a granular assessment of differential impacts of the DIP reform.

## 5 Limitation

Several limitations should be noted. The given research was based on the data of one municipality, which can be considered a limitation. Moreover, solely inpatient records were used, and this could have created a selection bias and underestimated the system wide impact of the reform on diabetes-related service usage. Lastly, causal inference cannot be done without an independently formed control group.

## 6 Conclusion

The DIP reform was effective in restraining the total hospitalization costs and LOS, but the course of the OOP ratio was in sharp contrast to such efficiency benefits. Costs were dropped across the board and the major factor contributing to this was standardization of clinical protocols and financial discipline brought about by the DIP framework. Compared to URBMI patients, URRBMI patients registered a greater reduction, a situation that could have been a result of disparity in generosity of benefits and reimbursability policy between the two insurance plans. Los in contrast reduced more in TCM hospitals, which may indicate that the model of DIP is not designed structurally to align with personalized, labor-intensive nature of the traditional Chinese medicine services.

The financial impact on patients varied considerably across insurance schemes. Although UEBMI beneficiaries experienced a sizeable reduction in their OOP burden, URRBMI patients experienced a steep rise. The ever-increasing gap indicates discrepancies in coverage depth and reimbursement limits, indicating that the grouping logic of DIP has structural differences in cost-sharing impacts for insured populations with varying levels of coverage, which may weaken the mutual assistance function of the system. The climbing OOP ratio within TCM facilities indicates particular revenue models, dependence to proprietary Chinese medicines which have lower reimbursement rates and the very nature of this system that it is quite difficult to standardize the cost of herbal treatments.

Finally, although the DIP reform has played a critical role in bringing about efficiency, and reducing costs, it has also revealed notable inequities in insurance plans and types of providers. It means that reforms in payment only based on efficiency can potentially cause equity gaps. To mitigate this, the changes in policy in the future should put health equity at the center to ensure that the benefits of the reform are distributed more equitably. In addition, the follow-up investigation must combine quantitative and qualitative research to ensure the complete involvement of strategic maneuvers of insurers, providers and patients, who have to wear this new structure.

## Data Availability

The data analyzed in this study is subject to the following licenses/restrictions: The data that support the findings of this study are not readily available because it is from a public institution, institutional review is required. Requests to access these datasets should be directed to Yiming Sheng, 202312210610019@zcmu.edu.cn.
